# Influence of socioeconomic status on antipsychotic prescriptions among youth in France

**DOI:** 10.1186/s12888-017-1232-3

**Published:** 2017-02-28

**Authors:** Olivier Bonnot, Mélanie Dufresne, Paula Herrera, Emmanuelle Michaud, Jacques Pivette, Anicet Chaslerie, Anne Sauvaget, Caroline Vigneau

**Affiliations:** 1 Department of Child and Adolescent Psychiatry, University Hospital of Nantes, Nantes, France; 2 Department of Pharmacology, University Hospital of Nantes, Nantes, France; 3 Medical Department, French National Health Insurance (DRSM), Nantes, France; 4 Grupo de Investigación en Neurociencias NeURos, Universidad del Rosario, Bogota, Colombia; 5 Department of Psychiatry, University Hospital of Nantes, Nantes, France; 6 Department of Child and Adolescent Psychiatry, University of Nantes, CHU de Nantes, 55 rue St Jacques, 44 000 Nantes, France

## Abstract

**Background:**

Recent studies analysing the trends in antipsychotic (AP) prescriptions for children and adolescents have raised concerns regarding the influence of socioeconomic status. Previous findings have also shown variable prescription rates for first-generation (FG) and second-generation (SG) APs.

**Method:**

Our objectives were to assess the proportion of patients from low-income families receiving APs and the most commonly prescribed APs in France. We conducted a descriptive analysis of AP drugs dispensed during a 1-year period (July 1, 2013–June 30, 2014) in a northwestern region of France with 941,857 subjects less than 18 years old. All data were extracted from an exhaustive, individual and anonymous social security database. We obtained each subject’s socioeconomic status (by identifying their affiliation with a specific social security program) and also collected sociodemographic data, drug type, prescribing and dispensing dates and amount, and prescriber type (e.g., hospital physician, general practitioner, psychiatrist, paediatrician).

**Results:**

There were two main novel findings. First, we found that the proportion of patients with AP prescriptions was nearly ten times higher in low-income families than in the general population: 35.9% of CMU-C patients compared to 3.7% in all of Pays de la Loire (*X* ^2^ = 7875.1, *p* < 0.001). Additionally, we found a higher rate of FGAP than SGAP prescriptions (65% vs. 57%).

**Conclusions:**

Our study suggests two types of AP misuse that could provide interesting targets for public healthcare interventions.

First, our results strongly suggest an over-representation of patients from low-income families. Low-income families primarily resided in areas with low physician density and appeared to receive drugs to treat their conditions more frequently than other individuals. This increased prescription rate is a public health issue, potentially requiring political action. Second, the use of FGAPs did not adhere to the latest recommendations for drug use in this population, and this discrepancy should be addressed with informational campaigns targeted to medical practitioners.

## Background

Antipsychotic (AP) drugs are recommended for the treatment for schizophrenia [1] and bipolar disorders [2] in children and adolescents. These drugs are effective in managing the core symptoms of these two diseases [1, 2]. APs are also partly effective and thus prescribed to manage attention deficit hyperactivity disorders, disruptive behaviour disorders, Tourette’s syndrome, post-traumatic stress disorder, anorexia nervosa and autism spectrum disorders [3, 4]. The recent literature regarding the prescription of APs raises two main issues:

The first pertains to the use of first- and second-generation APs. Considering the data on their efficacy and side effects, the literature recommends a first-line prescription of a second-generation AP (SGAP) and then switching to another SGAP if necessary before considering a first-generation AP (FGAP) [1]. The choice of prescription should balance the benefits and risks of the drug, particularly in younger populations, who experience more side effects than adults [5, 6]. *A recent study showed that AP treatment is associated with increased body weight and cardiometabolic side effects as well as different side effect patterns* [5]. The use of SGAPs is now recommended, and longitudinal studies have shown an increase in AP prescriptions in the last two decades with a switch from FGAPs to SGAPs [7–9]. However, some European studies, including those in France, have found that FGAPs are still much prescribed in this population, whereas in the U.S., the rate of FGAP prescriptions among youth is now very low [4, 10].

The second issue is related to the patients’ socioeconomic status (SES). Very few studies have explored the influence of socioeconomic/residential status on AP prescription rates. Existing research has focused on rural vs. urban inhabitants [11, 12] and has found no correlations between these parameters and AP prescriptions. Two recent studies on this topic yielded conflicting results. One study reported that AP prescriptions were more common among people from low-income areas in the UK [13], whereas the second study failed to find an association between AP consumption by youth and the rate of welfare benefits [14]. These inconsistent findings may have occurred because the authors correlated the overall rate of AP prescriptions with welfare benefits of geographic locations rather than individual welfare data. The effect of SES might be more related to individual factors than to the geographic location.

To study these two issues, we conducted a descriptive analysis of drugs dispensed during a 1-year period (July 1, 2013–June 30, 2014) in an exhaustive study of a northwestern region of France with 3,658,000 inhabitants, including 941,857 subjects younger than 18 years old whose socioeconomic conditions were known.

## Methods

### Data source and population sample

We conducted an observational study of a retrospective cohort using a healthcare database that included information on subjects living in one area (Pays de la Loire) with five different counties (Loire Atlantique, Maine et Loire, Mayenne, Sarthe, and Vendée).

The data were extracted from one database (datamart de Consommation Inter Regimes, DCIR) that was part of the French healthcare insurance database (Système National d’Information Inter Régimes de l’Assurance Maladie, SNIIR-AM), which contains all of the information from the main insurance schemes in France; the SNIIR-AM covers salaried workers, farm workers, non-salaried workers (i.e., craftsmen and tradesmen), and a few other special groups (i.e., teachers, policemen, and armed forces). Affiliation with one of these schemes is mandatory (i.e., persons cannot be outside of the database). The DCIR database is an exhaustive, individual and anonymous healthcare database that includes all information on refunded healthcare claims. Individuals cannot purchase any of the included drugs without a pharmacist’s report of this prescription being documented in the DCIR. Therefore, it is the main point of access for information on drug consumption by a large group of individuals. As with all subjects, individuals younger than 18 years have their own anonymous health service code, which is a lifetime code that uniquely identifies each resident.

Our population sample was composed of all children and adolescents younger than 18 years old at the end of the study period (July 1, 2013, to June 30, 2014) who were living in the Pays de la Loire area (northwestern France, 3.658 million inhabitants in 2013 [15]). All characteristics of the youth who received at least one dispensation of psychotropic drugs were obtained (*n* = 17.198).

### Psychotropic drug selection process in the database

Drugs were identified using the International Non-proprietary Name (INN) and categorized using the Anatomical Therapeutic Chemical (ATC) classification system [16], which is commonly applied in epidemiologic studies. We selected APs, which are a subcategory of N05, the N05A group. We also added valproic acid (N03AG01) and valpromide (N03AG02). The total list of drugs included was as follows: Chlorpromazine, Levomepromazine, Cyamemazine, Valproic acid, Valpromide, Periciazine, Pipamperone, Haloperidol, Flupentixol, Zuclopenthixol, Pimozide, Loxapine, Clozapine, Olanzapine, Quetiapine, Sulpiride, Tiapride, Amisulpride, Risperidone, Aripiprazole, and Paliperidone.

Valproic acid and valpromide are commonly used for their AP effects by prescribers in general practice in France (even if they are off-label), and we chose to include them after a consensus meeting with all the authors of this article (i.e., OB, CC, PH, CV, PJ).

### Study measures

We collected information on participants’ sociodemographic data (gender, county, affiliated healthcare insurance scheme, age, and CMU-C status). *Couverture Maladie Universelle Complémentaire* (CMU-C*)* is a supplemental universal public insurance coverage scheme for low-income persons. The criteria for accessing the CMU-C include an income less than 721 euros/month for a single individual, adding 250 euros for each member of the family. Affiliation with CMU-C is considered a poverty threshold in France. Therefore, CMU-C status defines the group of people under the poverty threshold in France.

We created 5 age categories: 0–4, 5–9, 10–14 and 15–17 years old.

Regarding medications, we collected the type of drug dispensed (INN and ATC code (5th level)), the date of dispensation, the date of prescription, the number of dispensations, and the prescriber type (e.g., hospital physician, general practitioner, psychiatrist, paediatrician).

Our database granted access to all drugs dispensed and reimbursed in a pharmacy (and thus all prescriptions). We were able to determine how many different dispensations each patient had received during the study period. In France, drugs are provided to patients in complete blister cards (with a pre-defined number of slots), not by pills. Patients are usually given 1 month of treatment per dispensation (with the number of complete blister cards calculated accordingly). Therefore, patients typically receive extra pills, which may represent more than a 1-month supply. We determined the treatment duration for each subject by accessing the number of different months of dispensation in the follow-up for each drug. The month of dispensation was obtained to determine whether treatment was chronic (continuous variable), and we considered subjects receiving at least 6 months of dispensations as chronic users, which was consistent with the methodology of a previous study using this database [14].

Finally, it was difficult to determine the type of hospital practitioner prescribing APs. All prescriptions from physicians working in a hospital are dispensed with the unique identification number of the hospital. Currently, the only way to access information on the type of hospital practitioner is to access the actual prescription, which would have provided the name and medical speciality of the physician; however, this approach was not possible in our study for ethical reasons (lack of anonymity).

We also searched for national data regarding the general population. We focused on the number of inhabitants [15], proportion of CMU-C patients [17] and medical density in the study region [18].

### Statistical analysis

Statistical analyses were performed using SAS Enterprise software version 4.3, and the detailed data were extracted and analysed by individuals who manage the healthcare insurance database according to the national commission for liberty and informatics (CNIL) laws.

A descriptive analysis was performed for sociodemographic and drug prescription data. We also calculated the proportion of AP prescriptions in comparison with general population data.

A proportions test based on chi2 was performed to compare the prevalence of CMU-C patients in our sample with that of the general population.

## Results

### Prescription overview

Among all patients in the Pays de la Loire region, 0.33% (2,897 children and adolescents under 18 years old) had an AP prescribed and dispensed during the observation period (see Table 1). Half of these prescriptions were for patients aged 13 years or younger (48%). Prescriptions increased with age and were higher in boys than in girls (0.47 in boys and 0.18 in girls).

**Table 1:** Raw 2013 prevalence rates of antipsychotic delivery by age and sex in Pays de la Loire and its 5 counties

Gender	Age (years)	Loire Atlantique		Maine et Loire		Mayenne		Sarthe		Vendée		Pays de la Loire (All)	
		*n*	% Pop	*n*	% Pop	*n*	% Pop	*n*	% Pop	*n*	% Pop 85	*n*	% Pop
MALE	0–4	4	0.01	12	0.05	9	0.09	4	0.02	-	-	29	0.02
	5–9	89	0.21	105	0.39	104	0.98	35	0.19	29	0.14	362	0.30
	10–14	221	0.52	233	0.89	183	1.75	114	0.63	123	0.62	874	0.74
	15–17	216	0.88	170	1.14	114	1.93	99	0.93	108	0.98	707	1.05
	Total	530	0.35	520	0.55	410	1.10	252	0.38	260	0.36	1972	0.47
FEMALE	0–4	5	0.01	4	0.02	2	0.02	3	0.02	1	0.01	15	0.01
	5–9	13	0.03	25	0.10	15	0.15	15	0.08	11	0.06	79	0.07
	10–14	61	0.15	97	0.38	52	0.52	33	0.19	23	0.12	266	0.24
	15–17	113	0.48	99	0.68	44	0.80	61	0.58	57	0.53	374	0.57
	Total	192	0.13	225	0.25	113	0.32	112	0.18	92	0.13	734	0.18
ALL	0–4	9	0.01	16	0.03	11	0.05	7	0.02	1	<0.01	44	0.02
	5–9	102	0.12	130	0.25	119	0.57	50	0.14	40	0.10	441	0.19
	10–14	282	0.34	330	0.64	235	1.15	147	0.41	146	0.38	1140	0.50
	15–17	329	0.68	269	0.91	158	1.38	160	0.75	165	0.75	1081	0.82
	Total	722	0.24	745	0.40	523	0.72	364	0.28	352	0.25	2706	0.33

Our results also showed that the overall AP prescription rate differed by county. Two counties were above the average prescription rate (0.33%): Mayenne (0.72%) and Maine et Loire (0.40%). Some rates, such as AP prescriptions for boys 5 to 9 years old in Mayenne vs. all areas (0.98 compared to 0.30), were of particular importance. The results in Mayenne were higher than the average for all sex and age subgroups; for example: 0.52 vs. 0.24% for girls aged 10–14 years and 1.75 vs 0.74% for boys aged 10–14 years. We present AP prescription rates by county with medical density in Table 2 (using data from the Conseil National de l’Ordre des Medecins) [18].

**Table 2:** Medical facility density (/100 000 inhabitants) and raw antipsychotic prescription rate in all Pays de la Loire department (*n* = 3 658 000) from July 1st 2013 to June 30th 2014

	Mayenne	Vendée	Sarthe	Maine et Loire	Loire Atlantique
Medical practionner density	179.8	201.3	212	283.7	308.3
Rate of AP prescriptions	0.72	0.25	0.28	0.40	0.24

### CMU-C-affiliated population

The proportion of individuals affiliated with CMU-C in our study was approximately 1/3 of our population (35.9%). When compared to the rates in the general population in Pays de la Loire, Chi^2^ comparison tests showed a higher proportion of CMU-C patients in our sample (*X* ^2^ = 7875.1, *p* < 0.001). The results were similar in all counties (see Table 3). CMU-C affiliation was higher in families with children over 5 years: 22.7% for ages 0–4, 36.3% for ages 5–9, 35.5% for ages 10–14 and 36.7% for ages 15–17 years.

**Table 3:** Patients with CMU-C in our sample and in general population

	Patients with CMU-C affiliation (*n*)	Patients with NO CMU-C affiliation (*n*)	Total	Proportion of individuals with CMU-C in general population (%)	95% CI/Chi2	*p*
Total Pays de la Loire Region	972	1734	2706	3.7	0.34–037/7875.1	<0.001
Loire Atlantique	250	472	722	4.6	0.31–0.38/1476.4	<0.001
Maine et Loire	255	490	745	4.9	0.31–038/1368.8	<0.001
Mayenne	190	333	523	4.2	0.32–0.40/1333.8	<0.001
Sarthe	132	232	364	6.2	0.31–041/560.5	<0.001
Vendée	122	230	352	3.1	0.29–0.39/1156.6	<0.001

Comparison test with chi2. All p are 0.20 10^−16^

### AP-specific medication data

Regarding single prescriptions, FGAPs were prescribed more frequently than SGAPs (65% vs. 57%). The ratio of FGAPs to SGAPs decreased with age, at 86% vs. 32% for subjects 0–4 years old and 63% vs. 61% for subjects 15–17 years old. In our study, 23% of subjects received both FGAPs and SGAPs. The overall data for all prescriptions (single and combination) are presented in Table 4.

**Table 4:** Type of antipsychotic and thymoregulator prescribed by age in all Pays de la Loire areas (*n* = 3 658 000) from July 1, 2013 to June 30, 2014

	Age								Total *n* = 2706	
	0–4 y *n* = 44		5–9 y *n* = 441		10–14 y *n* = 1140		15–17 y *n* = 1081			
Antipsychotic type and combination	*n*	%	*n*	%	*n*	%	*n*	%	*n*	%
SGAP single prescription	6	13.6	133	30.2	392	34.4	369	34.1	900	33.3
FGAP single prescription	36	81.8	237	53.7	447	39.2	390	36.1	1110	41.0
FGAP (only usedTourette)	-	-	5	1.1	3	0.3	3	0.3	11	0.4
Valproate or Valpromide	-	-	3	0.7	8	0.7	19	1.7	30	1.1
SGAP + FGAP	2	4.5	62	14.1	262	23.0	252	23.3	578	21.5
SGAP+ Valproate or Valpromide	-	-	1	0.2	8	0.7	8	0.8	17	0.6
FGAP+ Valproate or Valpromide	-	-	-	-	2	0.2	8	0.8	10	0.3
Various combination	-	-	-	-	18	1.2	32	3.0	50	1.8
FGAP/SGAP ratio	86.3/18.1		68.9/44.5		63.2/58.6		60.3/58.2		65.1/57.1	

*SGAP* second generation antipsychotic, *FGAP* first generation antipsychotic, *Tourette* AP used exclusively in Tourette syndrome in France (pimozide)

TGAP/SGAP ratio encompass all FGAP or SGAP, even if it is co-prescription (related to an amount of prescription more then a number of individuals)

The most commonly prescribed APs were cyamemazine and risperidone, totalling 1,379 and 1,303 prescriptions, respectively. Aripiprazole was the third-most prescribed AP, with only 207 prescriptions.

Regarding the duration of cyamemazine treatment, 33% of children/adolescents (463 of 1,379) had only a 1-month dispensation, and 27% (366 of 1 379) had 6 separate months with one or more dispensations per year. For risperidone, 18% of children/adolescents (230 of 1,303) had only one month of dispensed drugs, and 41% (532 of 1,303) had 6 separate months with one or more dispensations per year.

Regarding the type of prescribers, hospital physicians comprised 73.2% of the total. For 46.3% of the patients, these doctors were their only prescriber. General practitioners prescribed 11.4% of the patients’ prescriptions during the study period (results are in Table 5).

**Table 5:** Type of prescribers (alone or associated with others) in all Pays de la Loire regions (*n* = 3 658 000) for antipsychotic prescriptions from July 1, 2013 to June 30, 2014

*Specialty*	*Only prescriber for a particular patient*		*Other prescribers are in charge of the patient*		*Total n = 2706*	
	*n*	*%*	*n*	*%*	*n*	*%*
*Hospital any type (mostly paediatricians and psychiatrists)*	*1252*	*46.3*	*729*	*26.9*	*1981*	*73.2*
*General Practice*	*309*	*11.4*	*581*	*21.5*	*890*	*32.9*
*Psychiatrist*	*250*	*9.2*	*257*	*9.5*	*507*	*18.7*
*Paediatrician*	*15*	*0.6*	*26*	*1.0*	*41*	*1.5*
*Other*	*9*	*0.3*	*52*	*1.9*	*61*	*2.3*
*No Info*	*42*	*1.6*	*164*	*6.1*	*206*	*7.6*

Table is to read by line, and not per columns (for example 507 patients (18.7% of our 2 706 patient’s sample had prescription by a psychiatrist, among them 250 had only one prescriber, and 257 had more then one)

## Discussion

This study presented two main novel results. First, we found that the proportion of patients with APs was almost ten times higher in low-income families than in the general population: 35.9% of low SES patients compared to 3.7% in all of Pays de la Loire (*X* ^2^ = 7875.1, *p* < 0.001). In addition, we found a higher rate of FGAP than SGAP prescriptions (65% vs. 57%).

In general, our sample is comparable to those of other recent French studies, as our overall AP prescription rate was 0.33% (0.47 in boys and 0.18 in girls) for patients under 18 years old. Three major studies on the use of AP medications have been conducted using the French healthcare insurance database. In 2004, a study was conducted in two different regions, showing that 0.3% of patients used APs and that the rates increased with age [19, 20]. Using a different methodology and a random sample (1%) from the 2010 national database, another study reported an overall AP prescription rate of approximately 0.27–0.33% (0.35–0.45% for boys) [21]. A recent study examined a random representative sample of the French population (1%) extracted from the healthcare insurance database from 2006 to 2013 [14] and found an overall AP prescription rate of 0.49% for patients under 25 years old, 0.47% for patients between 11 and 15 years old, and 0.26% for patients between 6 and 10 years old.

These results are also consistent with recent studies in Europe. For example, a study in Germany used a similar method involving an exhaustive data source from a large area and found that the rate of AP prescriptions in 2012 was 0.33% and increased with age [13, 22]. Compared to older studies and those with different methodologies (smaller, non-exhaustive samples or random partial extractions of a large population), our results are within the mean range of data from Western countries, which show prevalences of APs ranging from 0.08% in Italy [23] to 0.76% in the U.S. [24]. Our rates in France are lower than those in the U.S., with the most recent French data reporting AP use in 2006 and 2010 by 0.14% and 0.11% of younger children, 0.85% and 0.80% of older children, 1.10% and 1.19% of adolescents, and 0.69% and 0.84% of young adults [21].

### Socioeconomic status

The results regarding SES are important and similar in all counties (see Table 3). To our knowledge, this type of data has not been presented in previous studies. Although these results differ from comparable French [14] and Canadian [12] studies, previous authors compared the global rate of low SES patients in an area to the AP rate, whereas our study included each parent’s CMU-C affiliation. Our approach provides a different perspective and suggests, by incorporating individual status, that being from a low-income family instead of simply living in a low-income area may be associated with increased prescription of APs.

In the same vein, we also found that the geographical distribution of AP users was not homogeneous and was greater in counties with the lowest medical density compared to those with a higher medical density (see Table 2). The ratio of medical facilities in the entire region was 272.7/100,000 inhabitants, which is slightly lower than the national density of 299.7/100,000 [18]. Moreover, the county with the lowest rate of AP prescriptions was Loire Atlantique, which also has the highest density of medical facilities. In contrast, the highest rate (0.72) was found in Mayenne, where the medical density is the lowest. These data suggest that individuals with greater medical access receive fewer AP prescriptions (see Table 2).

We found conflicting evidence in the literature regarding the impact of residence on AP prescriptions. A study from Taiwan found that AP prescriptions for patients aged 18 years or under were more frequent in suburban areas than in urban and rural areas [11], although this relationship was not found in a Canadian study [12]. However, Verdoux and colleagues found an increase in the level of AP prescriptions in areas with a low density of health facilities for children under 10 years old and in areas with low socioeconomic resources (global CMU-C affiliation rate in the area) for adolescents aged 16–20 years [14]. These conflicting results can be explained, again, by their use of data from large groups rather than from individually oriented data.

However, we could not determine a correlation between the density of medical facilities and access to health resources because of imprecisions in our data. Access to health care facilities depends on the *exact* part of the county where the patient resides (e.g., a family living in southern Mayenne may have better access to the University Hospital of Angers in Maine et Loire than some people in the extreme south of the Angers area). Another limitation of our study is that our data could not determine the purpose of the prescription or the patients’ diagnoses. For obvious privacy reasons, anonymous data prohibit access to these data in French insurance databases. Accordingly, we could not link AP prescriptions to specific diagnoses.

The higher level of AP prescriptions among children and adolescents from low-income families can be explained by two hypotheses:

1. Patients from low SES families are known to exhibit more psychiatric disorders, especially those associated with behavioural disturbances [25, 26]. This correlation is stronger for anxiety and mood disorders (which may be associated with behavioural disturbances in youth) and ADHD than for diseases such as autism, schizophrenia and Tourette’s syndrome [25, 26], which are core indications for AP prescription. Moreover, a higher incidence of behavioural problems has been found among preschool children from low SES families [27] and adolescents living in poverty [28]. Unfortunately, we were not able to verify this hypothesis, as we could not determine diagnoses in our sample.
2. The second hypothesis is related to the specific situations regarding child and adolescent psychiatry in France. A large percentage of child and adolescent psychiatrists in France do not commonly prescribe psychotropic medications, particularly APs. This practice is associated with the strong psychoanalytic culture in France along with a lack of current scientific knowledge about medications. Most of our colleagues consider themselves psychotherapists. Therefore, a psychiatrist may be more likely to prescribe medications for behavioural reasons than for developmental disorders (e.g., autism spectrum disorders, schizophrenia [29], Tourette’s). Additionally, there are few hospitalizations that occur for child and adolescent psychiatric disorders due to the limited number of beds in France for emergencies or short periods (13.5/100,000 inhabitants), especially in Pays de la Loire (7/100 000 in Loire Atlantique, 9 in Maine et Loire and 12 in Sarthe, with other counties having only long-term hospitalizations, which are more similar to boarding school) [30]. There is also a lack of non-medical psychiatric healthcare professionals such as clinical psychologists, as in contrast to psychiatrists, psychologists’ interviews are not reimbursed by social security [15].

These hypotheses may be synergistic. APs are frequently used to treat behavioural issues and may provide a quick solution to complex situations that would benefit from individual and family evaluation and support. When patients from low SES families exhibit more behavioural disorders, they encounter psychiatrists with few therapeutic options other than medications. Patients from higher SES families may be more likely to seek psychologists in private practice or re-educators (whose visits are not free).

Further studies that evaluate the specific level of care (medical and social) and the exact purpose for AP prescriptions are necessary to confirm this hypothesis.

### *FGAPs* vs. *SGAPs*

The rates of FGAP compared to those of SGAPs were particularly high among younger patients: 86% vs. 32%, respectively, for ages 0–4 years and 69% vs. 44%, respectively, for ages 5–9 years. Remarkably, the rates remained high in children 10 to 13 years old (39.2% FGAP vs. 34.4% SGAP) and adolescents from 15 to 17 years old (36.1% FGAP vs. 34.1% SGAP). Indeed, recent AMMs (Market Release Authorizations) for SGAPs have made them available to 13- to 15-year-old patients instead of only 18-year-old patients (for aripiprazole, olanzapine and risperidone). Moreover, in 2007, an AMM was obtained for the use of risperidone in 5-year-olds for behavioural disorders involving intellectual deficiency. Therefore, SGAPs could have been prescribed more extensively.

Our results indicate higher rates of FGAP than those reported in the most recent comparable studies in France [14, 31] for all ages, with rates of FGAPs vs. SGAPs for infants, children and adolescents of 86.3% vs. 53.6%, 68.9% vs. 41.5% and 83.9% vs. 58.8%, respectively [14]. However, these previous studies involved a representative national sample that did not reflect regional disparities (see explanation for Pays de la Loire above).

This high level of FGAP prescription may be harmful because the side effect profile of APs differs drastically, especially in the paediatric population [6, 32]. In other countries, longitudinal studies have shown an increase in AP prescriptions in the last two decades as well as a switch from FGAPs to SGAPs [7–9].

The high use of cyamemazine, which was the most commonly prescribed AP in our study, is likely related to French habits. In France, cyamemazine is mainly used as a sedative and anxiolytic drug, despite its poor tolerance due to metabolic syndrome as well as its limited AP effects [33]. Cyamemazine was also originally developed by a French laboratory and was marketed heavily in the 1990s. However, cyamemazine has a low affinity for dopaminergic receptors and therefore poor AP activity. The sedative effects of cyamemazine are mediated through its 5HT2A activity [34]. The main scientific literature regarding cyamemazine has investigated its efficacy in treating benzodiazepine withdrawal, particularly for alcoholic patients (for a review, see [35]). The very high level of cyamemazine use is likely related to a lack of knowledge of the efficacy and side effects as well as to prescribers’ habits. The lack of knowledge about new SGAPs and their AMMs for younger patients may also be a reason for the low level of SGAP prescription, particularly during adolescence. Our results are comparable with those of previous studies showing a high rate of cyamemazine use in France [14].

## Conclusion

Our epidemiological study in an exhaustive population demonstrated that the consumption of APs is concerning, occurring in 33 children and adolescents out of 10,000. This overall rate is admissible, but the qualitative analysis suggests a few concerning findings that should be addressed.

There was a misuse of APs, with an excessive number of FGAP prescriptions, especially for cyamemazine. This practice appears to be related to a lack of knowledge regarding the efficacy and side effects of this drug. The FGAP/SGAP ratio in northwest France was higher than the national mean [14] and was not compliant with the latest recommendations for drug use in this population. This discrepancy should be addressed through an information campaign directed towards medical practitioners and in the teaching of medical students. As our work was conducted with the French National Health Insurance organization, a full partnership to undertake this type of campaign is already in place.

Despite the lack of information regarding the reasons for prescription, our results strongly suggest an over-representation of patients from low SES families in our sample of youth receiving APs. These patients were mainly living in areas with a low physician density and received drugs to treat their conditions more often than other individuals. Therefore, it is important to communicate these findings to medical practitioners, and political action to address these discrepancies may be necessary.

## Abbreviations

ADHD: Attention deficit hyperactivity disorder
AP: Antipsychotic
ATC: Anatomical therapeutic chemical
BP: Bipolar disorder
CNIL: Commission Natioanale Informatique et Liberté = national commission for liberty and informatics
DCIR: Datamart de Consommation Inter Regimes
EMA: European medicine agency
ESCAPAD: Enquete sur la Santé et les Consommations lors de l’appel de la Preparation à la Defense Nationale = survey on health and consummation during army conscription mandatory day
FDA: Food and drug administration
FGAP: First generation antipsychotic
INN: International non-proprietary name
SGAP: Second generation antipsychotic
